# Simulating long-term wildfire impacts on boreal forest structure in Central Yakutia, Siberia, since the Last Glacial Maximum

**DOI:** 10.1186/s42408-023-00238-8

**Published:** 2024-01-04

**Authors:** Ramesh Glückler, Josias Gloy, Elisabeth Dietze, Ulrike Herzschuh, Stefan Kruse

**Affiliations:** 1 Polar Terrestrial Environmental Systems, Alfred Wegener Institute Helmholtz Centre for Polar and Marine Research, Telegrafenberg A45, Potsdam, 14473 Germany; 2 Institute of Environmental Science and Geography, University of Potsdam, Karl-Liebknecht-Strasse 24-25, Potsdam, 14476 Germany; 3 Faculty of Environmental Earth Science, Hokkaido University, N10W5, Sapporo, 060-0810 Japan; 4 Institute of Geography, Georg-August-University Göttingen, Goldschmidtstrasse 5, Göttingen, 37077 Germany; 5 Institute of Biochemistry and Biology, University of Potsdam, Karl-Liebknecht-Strasse 24-25, Potsdam, 14476 Germany

**Keywords:** Individual-based, IBM, Fire regime, Larch, *Larix*, Modeling, LAVESI, Russia, Sakha

## Abstract

**Background:**

Wildfires are recognized as an important ecological component of larch-dominated boreal forests in eastern Siberia. However, long-term fire-vegetation dynamics in this unique environment are poorly understood. Recent paleoecological research suggests that intensifying fire regimes may induce millennial-scale shifts in forest structure and composition. This may, in turn, result in positive feedback on intensifying wildfires and permafrost degradation, apart from threatening human livelihoods. Most common fire-vegetation models do not explicitly include detailed individual-based tree population dynamics, but a focus on patterns of forest structure emerging from interactions among individual trees may provide a beneficial perspective on the impacts of changing fire regimes in eastern Siberia. To simulate these impacts on forest structure at millennial timescales, we apply the individual-based, spatially explicit vegetation model LAVESI-FIRE, expanded with a new fire module. Satellite-based fire observations along with fieldwork data were used to inform the implementation of wildfire occurrence and adjust model parameters.

**Results:**

Simulations of annual forest development and wildfire activity at a study site in the Republic of Sakha (Yakutia) since the Last Glacial Maximum (c. 20,000 years BP) highlight the variable impacts of fire regimes on forest structure throughout time. Modeled annual fire probability and subsequent burned area in the Holocene compare well with a local reconstruction of charcoal influx in lake sediments. Wildfires can be followed by different forest regeneration pathways, depending on fire frequency and intensity and the pre-fire forest conditions. We find that medium-intensity wildfires at fire return intervals of 50 years or more benefit the dominance of fire-resisting Dahurian larch (*Larix gmelinii* (Rupr.) Rupr.), while stand-replacing fires tend to enable the establishment of evergreen conifers. Apart from post-fire mortality, wildfires modulate forest development mainly through competition effects and a reduction of the model’s litter layer.

**Conclusion:**

With its fine-scale population dynamics, LAVESI-FIRE can serve as a highly localized, spatially explicit tool to understand the long-term impacts of boreal wildfires on forest structure and to better constrain interpretations of paleoecological reconstructions of fire activity.

**Supplementary Information:**

The online version contains supplementary material available at 10.1186/s42408-023-00238-8.

## Resumen

**Antecedentes:**

Los incendios de vegetación son reconocidos como un componente importante de los bosques boreales dominados por alerces en el este de Siberia. Sin embargo, la dinámica a largo plazo entre fuegos y vegetación en este ambiente único es pobremente entendido. Estudios paleo-ecológicos recientes sugieren que la intensificación de regímenes de fuego puede inducir cambios, a escala de milenos, en la estructura y composición de estos bosques. Estos pueden, a su vez, resultar en una retroalimentación positiva en la intensificación de los incendios y en la degradación del permafrost, aparte de amenazar la vida humana. Los modelos de fuego-vegetación más comunes, no incluyen específicamente detalles basados en la dinámica poblacional de árboles individuales, aunque el enfocarnos en los patrones de la estructura forestal que emerge de interacciones entre árboles individuales puede proveer de una perspectiva beneficiosa sobre los impactos de los cambios en los regímenes del fuego en el este de Siberia. Para simular estos impactos en la estructura forestal a escalas de milenios, aplicamos el modelo espacialmente explícito, individualmente basado, LAVESI-FIRE, expandido en un nuevo módulo de fuego. Observaciones de fuego basadas en imágenes satelitales junto con datos de campo fueron usados para informar la implementación de la ocurrencia de fuegos de vegetación y ajustar los parámetros del modelo.

**Resultados:**

Las simulaciones del desarrollo anual de los bosques y de la actividad de los incendios en un sitio de estudio de la República de Sakha (Yakutia) desde el Último Máximo Glacial (c. 20.000 años atrás), resaltan los impactos variables de los regímenes de fuegos en la estructura forestal a través del tiempo. La probabilidad modelada de forma anual en la ocurrencia de incendios y subsecuentemente del área quemada durante el Holoceno se compara muy bien con la reconstrucción del flujo de carbón en los sedimentos lacustres. Los incendios de vegetación pueden verse mediante diferentes patrones de regeneración del bosque, dependiendo de la frecuencia e intensidad de los fuegos y de las condiciones del bosque en el pre-fuego. Encontramos que fuegos de mediana intensidad a intervalos de retorno de unos 50 años o más, benefician la persistencia del alerce dahurian (*Larix gmelinii* (Rupr.) Rupr.), especie resistente al fuego, mientras que los fuegos de reemplazo tienden a permitir el establecimiento de coníferas siempreverdes. Aparte de la mortalidad post-fuego, los fuegos de vegetación modulan el desarrollo del bosque, fundamentalmente a través de efectos de competencia y una reducción de la capa de mantillo predicho por el modelo.

**Conclusiones:**

Con sus características de detectar la dinámica de la población a una escala fina, el modelo LAVESI-FIRE puede servir como una herramienta altamente localizada y espacialmente explícita, para entender los impactos a largo plazo de los bosques boreales sobre la estructura forestal y para mejorar los condicionamientos de las interpretaciones de las reconstrucciones paleo ecológicas en la reconstrucción de la actividad de los incendios.

## Background

Eastern Siberia has experienced extreme wildfire seasons in recent years (Hayasaka 2021; Ponomarev et al. 2023). Despite wildfires being an essential ecological process of the larch-dominated boreal forest (Kharuk et al. 2021), there is growing concern that a continued increase in fire activity may compromise the resilience of the forests, while at the same time threatening human health and safety (Reisen et al. 2015; Efimova et al. 2018). Due to the complexity of fire ecology, dependent on many interrelated variables, there is high uncertainty in any simulations of future local fire regime changes (Hantson et al. 2016). This is exacerbated by a lack of long-term information on fire regime changes and their impacts, especially in the eastern Siberian part of the boreal zone (Glückler et al. 2022).

Deciduous larch (*Larix* spp.)—Dahurian larch (*Larix gmelinii* (Rupr.) Rupr.), Cajander larch (*Larix cajanderi* Mayr), and Siberian larch (*Larix sibirica* Ledeb.)—account for the largest share of trees in eastern Siberia, their dominance being a remnant of the last glacial period (Herzschuh et al. 2016; Herzschuh 2020). Within the larch forests, other conifers can occasionally be found, for example, Siberian spruce (*Picea obovata* Ledeb.), Scots pine (*Pinus sylvestris* L.), or Siberian pine (*Pinus sibirica* Du Tour). By shedding their needles, larches growing in relatively dense stands add litter to an insulating organic layer, protecting deep permafrost grounds from accelerated degradation (Zhang et al. 2011; Herzschuh 2020). This deep permafrost explains why boreal eastern Siberia acts as an important carbon sink within the ecosystem-wide carbon budget of high latitudes (Watts et al. 2023). Dahurian larches are thought to possess pyrophytic properties (Tsvetkov 2004) and their accumulated litter of shed needles benefits occasional low-intensity surface fires. These fires help renew larch populations by opening the ground for seed germination while limiting invasion by evergreen conifers (Kharuk et al. 2021). However, increased fire intensity and/or frequency may interfere with this ecological balance and substantially change the structure of the forests, mitigating or even reversing their function as a carbon sink (Fan et al. 2023; Watts et al. 2023). It is expected that fire regimes in eastern Siberia will continue to intensify (Ponomarev et al. 2018; Talucci et al. 2022), but long-term impacts of fire regime changes on forest structure are poorly understood and depend not only on the immediate fire impacts, but also on the post-fire regeneration pathways of the forest.

In light of the unique interplay of larch-dominated forest, permafrost, and wildfires, the general understanding of long-term fire-vegetation interactions in eastern Siberia may benefit from including a highly localized and long-term perspective of individual trees, their life cycles, and competition for growth. Emergent patterns of individual-based tree population structure under wildfire stress may offer new insights into the consequences of currently intensifying fire regimes and also benefit any interpretations of reconstructed paleoecological fire records.

Efforts have been made in recent decades to include fire in dynamic global vegetation models (DGVMs; Hantson et al. 2016). However, due to their coarse grid-based globalized architecture, DGVMs generally cannot consider fine-scale interactions between different plant species, life cycles, population dynamics, and fire occurrence, nor consider the position, and thus related effects, of individual plants in the environment (Shuman et al. 2011; McKenzie et al. 2014). This may limit their analytical power in a region like eastern Siberia, where larch tree life cycles, successional patterns, and their interaction with the immediate physical environment are key ecosystem components, making the individual responses of the few dominant tree species to wildfires especially important to consider (Shuman et al. 2011).

Apart from DGVMs covering northern Eurasia, multiple fire-vegetation modeling studies have focused specifically on Siberia while following a variety of research questions and subsequent model setups. Ito (2005) simulated the carbon budget of wildfire occurrence in the boreal forest near Yakutsk throughout 1200 years with the model Sim-CYCLE, finding a mean fire return interval of 64 years and a predominantly surface fire regime, affecting 1.6% of the forested area per year. Applying SiBCLiM, Tchebakova et al. (2009) simulated the response of dedicated vegetation classes, permafrost, and fire occurrence to climate-change scenarios. In northern Siberia, they predict a tundra-to-forest change, whereas in southern Siberia and Central Yakutia an increase in wildfire activity may be followed by widespread steppe formation with higher tree mortality. Their results also suggest that, due to the resilience of permafrost, larches will remain dominant. To include the response of larches, Sato et al. (2010) applied an adapted version of the individual-based SEIB-DGVM to simulate post-fire forest recovery near Yakutsk. Using fixed scenarios of stand-replacing fire occurrence, their model considered explicit larch population dynamics. Zhang et al. (2011) used the dynamic vegetation model FAREAST, coupled with a permafrost model and expanded by the ability of stand-replacing wildfires to occur, to show that climate warming above c. 2 °C would impact species composition of the eastern Siberian larch forest, decoupling it from permafrost and possibly resulting in a forest state change towards dark taiga. An adapted version of FAREAST, the individual tree-based forest gap model UVAFME, was expanded by a more complex fire module by Shuman et al. (2017). Simulating scenarios with and without fire occurrence, they were able to show how wildfires generally benefit the more fire-adapted larch in its competition against other conifers. Finally, Stuenzi et al. (2022) simulated scenario-based disturbance effects, including fire scenarios, on tree populations and permafrost hydrology in the coupled LAVESI-CryoGrid model. Only in the surface fire scenario was the larch forest able to recover to pre-fire density, although the post-fire recovery was found to be linked to the moisture conditions of following years.

Apart from model-based studies, any paleoecological evidence for the long-term impacts of changing fire regimes on boreal vegetation remains sparse in eastern Siberia. Recent evidence from lake sediment analyses suggests potential positive feedback mechanisms between intensifying wildfire regimes and more open forests (Glückler et al. 2022), reinforcing the results of the modeling study by Tchebakova et al. (2009), while emphasizing the need for an improved understanding of fire regime changes on long timescales. Where paleoecological studies are lacking, long-term simulations in fire-vegetation models may contribute insights into forest responses to changing fire regimes.

We aim to contribute to a characterization of long-term impacts and regeneration patterns by introducing climate-driven fire disturbance to simulated tree population dynamics in the eastern Siberian boreal forest. To do so, we expand the individual-based, spatially explicit vegetation model LAVESI (Kruse et al. 2016, 2018, 2022a) with the ability to simulate variable wildfire regimes. The newly expanded model (LAVESI-FIRE) is used to simulate fine-scale, spatially explicit fire-vegetation dynamics over the last 20,000 years at a key study site in Central Yakutia, Siberia. This is complemented by investigating the effects of different fixed fire return intervals and fire intensities on tree density, stand ages, and species composition.

## Methods

### Study location

Central Yakutia, within the Republic of Sakha (Yakutia), the largest administrative unit in eastern Siberia, is characterized by its vast larch forests, underlain by continuous permafrost, and its common and culturally important thermokarst basin landforms (alaas; Crate et al. 2017, Fedorov 2022). The forest is dominated by the deciduous Dahurian larch. In between, there are patches of mixed forest with Siberian spruce and Scots pine. Within an alaas, the vegetation consists of grasses and sedges, whereas in the forest, ground vegetation is made up of mosses and lichens in larch needle litter and duff (comprising the organic layer; Sofronov and Volokitina 2010; Kruse et al. 2019b).

**Fig. 1:**
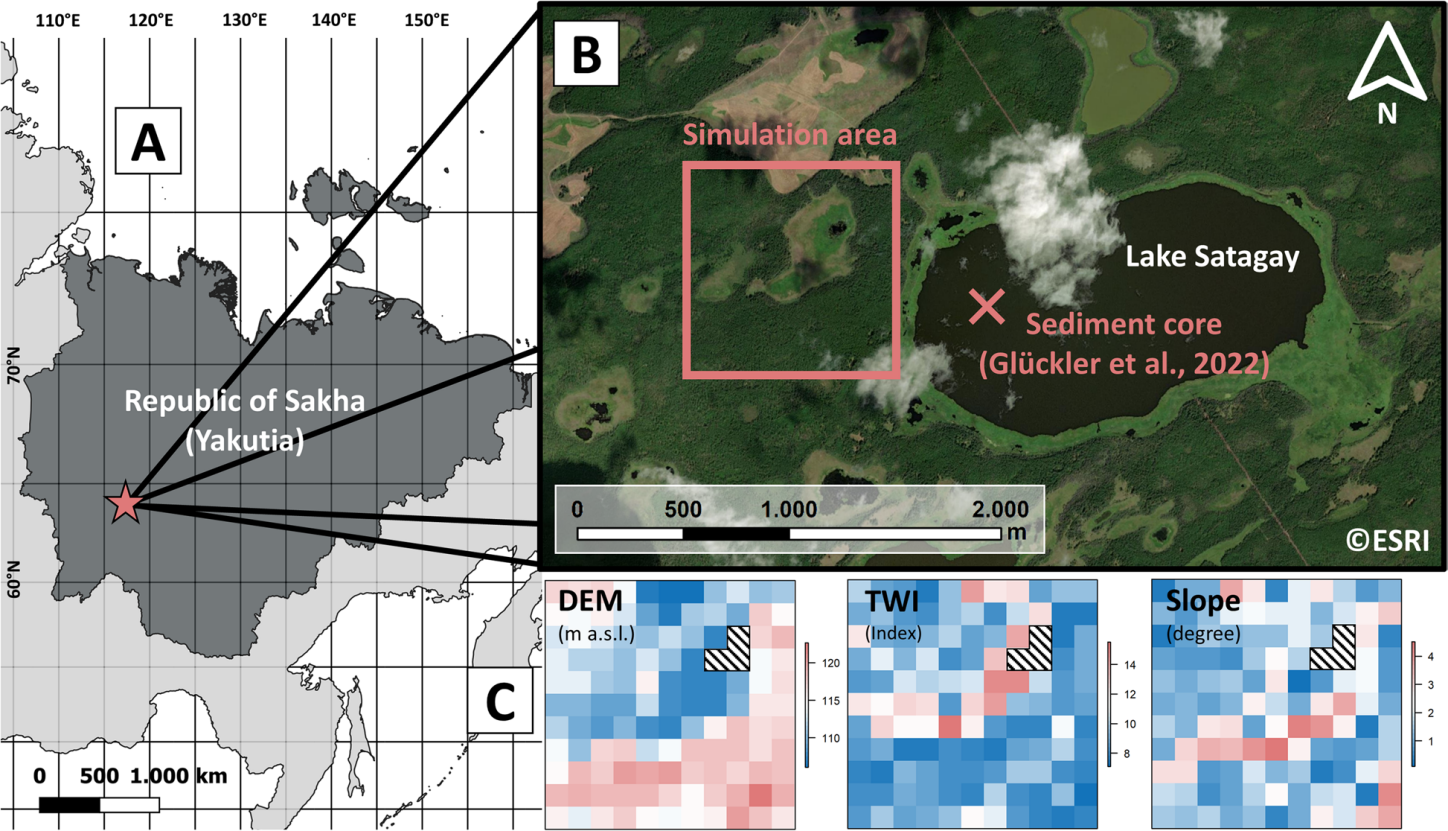
Map of the study area, including the location of the simulation area next to Lake Satagay. **A** Republic of Sakha (dark gray) within Russia (light gray) and the location of the study site (star symbol). **B** Satellite image of the study site, including the simulation area and the location of the sediment core used for a Holocene reconstruction of fire-vegetation interactions in Glückler et al. (2022). **C** Digital elevation model (DEM) and derived topographic wetness index (TWI) and slope for the simulation area. Hatched raster cells mark water bodies. Service layer credits: Esri, DigitalGlobe, GeoEye, i-cubed, USDA, USGS, AEX, Getmapping, Aerogrid, IGN, IGP, swisstopo, and the GIS User Community

The region is also known for its highly continental climate, experiencing short, warm summers and extremely cold winters. This results in a short vegetation period of 136 ± 6 days (mean ± 1σ), based on climate data from the Max Planck Institute Earth System Model 1.2 (MPI-ESM1.2) between 2000 to 2020 CE (Dallmeyer et al. 2022; Kleinen et al. 2023). The maximum amplitude between the warmest and coldest temperatures recorded within a single year can reach 100 °C. Based on CRU TS v4.06 data (Harris et al. 2020) at the study site near the town of Nyurba (Fig. 1), the mean annual temperature between 2012 and 2020 CE was − 7.1 °C, with mean monthly temperatures in January and July of − 32.3 and 17.8 °C, respectively. The mean annual precipitation sum was 303 mm. Mean monthly precipitation sums for January and July were 15 and 56 mm, respectively. The summer months of June, July, and August accounted for 47% of all annual precipitation.

Central Yakutia, especially west of the Lena River, experienced severe wildfire seasons in recent years (relative to years with available satellite observations). An evaluation of MODIS-derived burned area for 2001 to 2021 is provided by Glückler et al. (2022), showing that 2021 was the year with the largest burned area since 2001 within a 100 km^2^ buffer around the study site. Although fire regimes in eastern Siberia are generally described as low-intensity surface fires (Rogers et al. 2015), fires in 2021 were observed to engulf whole tree stands and threaten settlements, including Nyurbachan, c. 30 km north of the study site. It is expected that, similar to many other regions, Central Yakutia will continue to experience severe wildfire seasons, among other disturbances, with continued climate change (de Groot et al. 2013, Kukavsykaya et al. 2013, Sayedi et al. 2023[Preprint]).

In this western part of Central Yakutia, Lake Satagay (63.078°, 117.998°; 114 m a.s.l.) recently served as a study site for sedimentary paleoecological studies. The thermokarst lake, formed during the Late Glacial (between the Last Glacial Maximum and the Holocene, c. 20,000 to 11,700 ka BP), was analyzed to obtain records of both lake development stages and lake ecology (Baisheva et al. 2023), as well as surrounding vegetation and wildfire activity (Glückler et al. 2022) throughout the past c. 10,800 years. Both studies describe the thermokarst lake’s surroundings in more detail. The region is representative of the typical landscape in Central Yakutia, which is why a simulation area close to the western shore of Lake Satagay was determined to serve as a location for long-term simulations in this study (Fig. 1).

### Model description

The model LAVESI-FIRE developed in this study is a modified version of the individual-based, spatially explicit vegetation model LAVESI (*Larix* Vegetation Simulator), written in C +  + . LAVESI was first conceived to model fine-scale population dynamics of larches at the northern tundra-taiga interface and to simulate the advance of the northern treeline in Siberia under a warming climate (Kruse et al. 2016, 2019a, 2022b; Wieczorek et al. 2017). A detailed description of the initial model, parameterization, and validation, as well as localization for the eastern Siberian boreal forest, was done by Kruse et al. (2016). A simulation with this model consists of a custom simulation area, including environmental information about, for example, a litter layer (where needle litter accumulation is simulated as a partial representation of the organic layer) or the active layer depth, and entities of individual trees and seeds, both in cones and on the ground, on a 0.2 × 0.2 m sub-grid. In this simulation area, individual trees can grow in explicit locations. LAVESI computes annual cycles of weather-, environment-, and competition-dependent tree growth; seed production and dispersal; establishment; aging; and mortality. Later additions to the original model code include the ability to simulate wind-driven seed and pollen dispersal (LAVESI-WIND; Kruse et al. 2018). In the process of coupling LAVESI with the multilayer permafrost model CryoGrid (Westermann et al. 2016), the catalog of tree species simulated within LAVESI was expanded to feature besides Dahurian larch also Cajander larch, Siberian larch, Siberian spruce, Siberian pine, and Scots pine (LAVESI-CryoGrid; Kruse et al. 2022a, Stuenzi et al. 2022). Additionally, an explicit representation of landscape was implemented, allowing the model to use information on elevation, slope, and surface moisture from a topographic wetness index (TWI), derived beforehand from a digital elevation model (DEM) of the simulation area (Kruse et al. 2022a). More recently, Gloy et al. (2023) implemented and tested the effects of variation and inheritance of traits such as the weight of seeds or the resistance to drought, whereas Kruse et al. (2023) applied the model to investigate the upslope advance of the mountainous treeline under different climate pathways. Stuenzi et al. (2022) applied the coupled LAVESI-CryoGrid model to simulate the impacts of scenario-based disturbances, among them wildfire scenarios, on the forest and underlying permafrost. Because wildfires were introduced in a specific surface and canopy fire scenario, we aim to build on these findings by implementing climate-driven, variable fire regimes within the simulated environment of LAVESI. LAVESI-FIRE is based on the version of LAVESI used for the coupled LAVESI-CryoGrid model, that is, it includes multiple tree species and an explicit environment, but in its current version is not coupled to CryoGrid and does not include trait inheritance.

### Wildfire module in LAVESI-FIRE

**Fig. 2:**
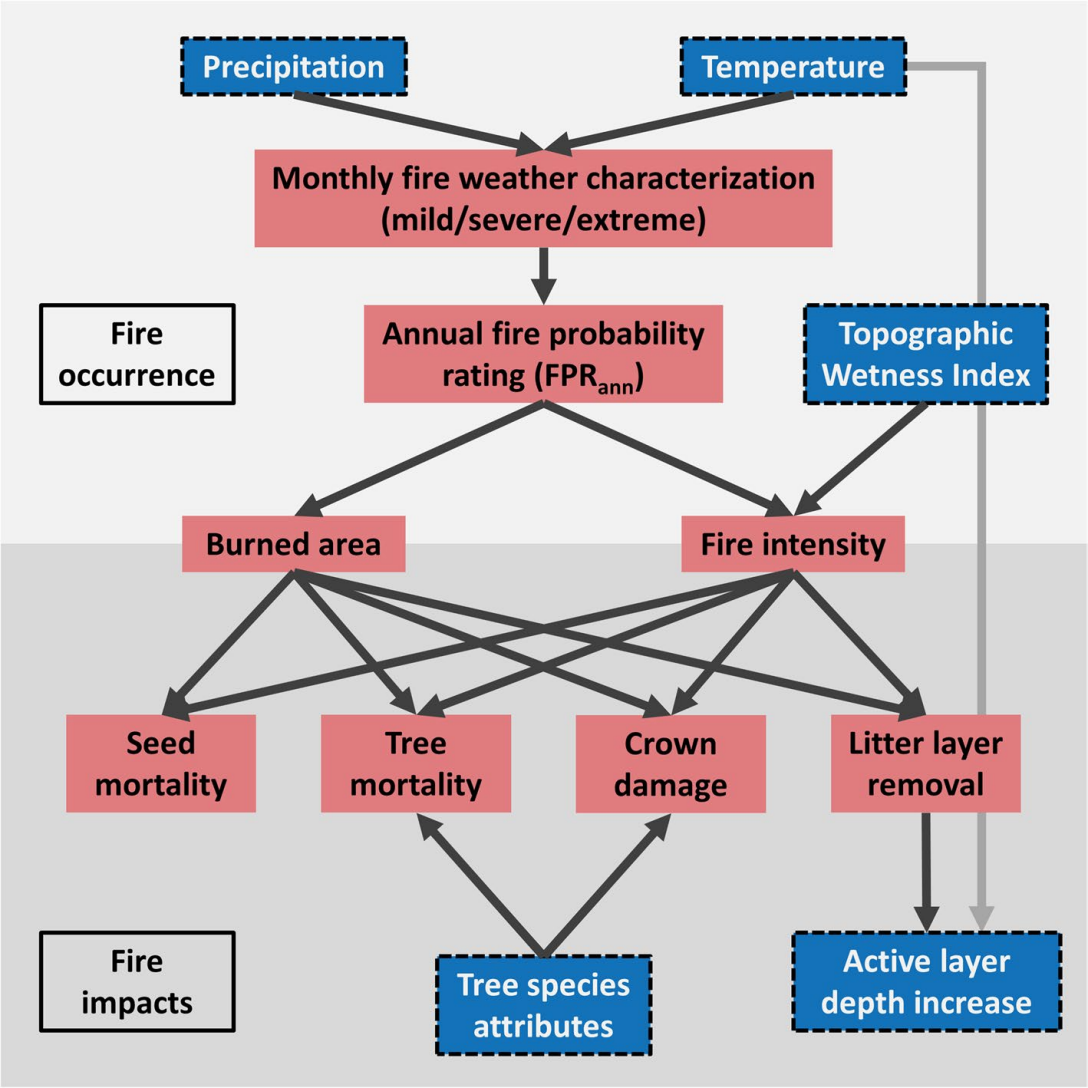
Conceptual diagram describing the new fire module within LAVESI-FIRE. Red/non-framed elements represent new components in the model, whereas blue/dash-framed elements were already present in previously published versions of LAVESI

LAVESI-FIRE simulates climate-driven fire occurrence within the simulation area, including fire impacts on trees and the environment. A wildfire can stochastically occur within each annual simulation step, with a probability dependent on monthly fire weather conditions (Fig. 2). Fire probability for each month (fire probability rating; FPR_mon_) is empirically estimated with a linear model of temperature (T) and precipitation (P; Supplement 1). This linear model was derived from a principal component analysis of the number of monthly MODIS-detected fire pixels in a 100-km radius around the study site (MCD64A1 product; Giglio et al. 2016), and T and P from weather station observation-based CRU TS v4.06 climate data for months above 0 °C (Harris et al. 2020), showing the best fit among several tested alternatives (*R*^2^ = 0.24; Supplement 2).

Values for FPR_mon_ are categorized and counted as either mild (*n*_mild_), severe (*n*_severe_), or extreme (*n*_extreme_) fire weather conditions. The threshold for an FPR_mon_ value to be categorized as *n*_mild_ was set as the highest FPR_mon_ value predicted for months in which no actual fires were detected by MODIS (Supplement 2). Thresholds for *n*_severe_ and *n*_extreme_ were set as third and fourth quantiles of the distribution of all possible FPR_mon_ values for the climate input at the study site, respectively (Supplement 3). For each year, FPR_mon_ categories are then summarized as an annual fire probability rating (FPR_ann_) between zero and one, directly representing ignition probability (Supplement 4). The calculation of FPR_ann_ was tuned to result in a mean FPR_ann_ = 0.03 for the climate input at the study site, or an average of one fire occurrence per c. 33 years, as a realistic value based on fire return intervals reconstructed in paleoecological studies in Yakutia (Glückler et al. 2021).

If an ignition takes place (i.e., if a randomly drawn uniform number between zero and one is below FPR_ann_), a random coordinate of the simulation area is chosen as the center. Around that center, a fire occurs, with an affected area (diameter) determined by FPR_ann_ relative to the width of the square-shaped simulation area. Fire intensity is estimated for each cell in the 0.2 × 0.2 m sub-grid of the fire-affected area, as the FPR_ann_ mediated by the local TWI (representing surface moisture availability; Supplement 5). Initially, both fire extent and intensity are thus linked to the fire weather conditions, which is supported by empirical evidence (Jones et al. 2022). Additionally, Glückler et al. (2022) demonstrated that around the study site extreme fire weather is well correlated with burned area. Fire impacts to vegetation in the model are, however, based on multiple local conditions and thus heterogeneous within a single fire-affected area.

Within this fire-affected area, trees, cones, seeds, and the litter layer can be affected in a variety of ways, based on important general fire impacts on vegetation (Wirth 2005; Hood et al. 2018; Bär et al. 2019; Kharuk et al. 2021). Tree mortality is directly affected by a combination of heat impacts to the stem (simulating cambium necrosis and xylem hydraulic failure) and/or damage to the canopy (simulating loss of foliage and buds, resulting in carbon starvation). The magnitude of both effects is decided by local fire intensity, which is directly linked to flame height (Rothermel and Deeming 1980; Heskestad 2016). These impacts on tree mortality can be mediated (or exacerbated) by species-specific and height-dependent traits such as insulating bark thickness and the ability to re-sprout (Wirth 2005; Schulze et al. 2012). These traits were introduced for different tree species by Kruse et al. (2022a). A tree growing within a sub-grid cell will be affected by that cell’s fire intensity alone, whereas for a tree growing on the border of multiple cells the mean fire intensity of all included cells is applied. Seeds in tree cones are removed depending on the local fire intensity, whereas seeds on the ground are always removed in cells with a fire intensity above zero (Kharuk et al. 2021). Finally, fire occurrence will also lead to a partial loss or complete removal of the litter layer (Delcourt et al. 2021). Previously introduced stochastic, small-scale disturbances of the litter layer and its regeneration at 0.5 cm year^−1^ (Kruse et al. 2022a) lead to an average height of c. 13 cm, corresponding with field observations of organic layer height (Kruse et al. 2019b).

Relevant post-fire processes, for example, a deepening of the active layer due to the removal of insulating litter and subsequent regeneration (Gorbachev and Popova 1996; Tchebakova et al. 2009; Knorre et al. 2019), succession of young trees on freshly cleared soil, or growth benefits for surviving trees due to a decrease in competition (Kharuk et al. 2021), are already part of vegetation dynamics within LAVESI (Kruse et al. 2016).

### Model inputs and simulation scenarios

The simulations were forced by monthly mean temperature (*T*_mon_) and monthly precipitation sum (*P*_mon_), extracted from a long-term global climate simulation with MPI-ESM1.2 (Dallmeyer et al. 2022; Kleinen et al. 2023). The simulation covers the years 24,900 to 0 years BP and ran with the spatial resolution T31 (c. 3.75° × 3.75° on a Gaussian grid) for the atmosphere and land component. We used the output for the grid cell that corresponds to the location of Lake Satagay. The climate timeseries was extended from 1950 to 2021 CE by appending the MPI-ESM1.2-HR and -LR (SSP126) climate data as featured in CMIP6 (O’Neill et al. 2016).

To test the sensitivity of simulated long-term trends of forest structure towards the climatic forcing input, we ran additional simulations with both climate data from a slightly different MPI-ESM model setup covering the time since 25,000 years BP (MPI-ESM-CR; Kapsch et al. 2022), as well as TraCE-21ka (“Transient Climate Evolution”) modeled climate data (22,000 years BP to 1990 CE; He 2011), both at the same spatial resolution of c. 3.75° and appended to 2021 CE in the previously described way. All individual climate timeseries were localized by fitting to means of *T*_mon_ and *P*_mon_ of the CRU TS v4.06 product (Harris et al. 2020) for the period of 1901 to 1949 (MPI-ESM1.2 and MPI-ESM-CR), 1901 to 2021 (combined MPI-ESM1.2-HR and -LR), and 1901 to 1990 CE (TraCE-21ka), respectively. For the randomly sampled wind forcing data of LAVESI-FIRE, six-hourly wind speed and direction data were obtained for Lake Satagay coordinates from the ERA 5 product (Hersbach et al. 2020) for the period from 2000 to 2020 CE.

For a one-at-a-time sensitivity analysis following Kruse et al. (2018), simulations were run with the main climate forcing data with and without the inclusion of the new wildfire module, and with *T*_mon_, *P*_mon_, and fire-induced tree mortality set to ± 5%, respectively.

Landscape input for simulations in this study was derived from the TanDEM-X 90 m digital elevation model product (DEM; Rizzoli et al. 2017). A simulation area of 990 × 990 m was used, set at the western shore of Lake Satagay (Fig. 1), with slope and a topographic wetness index (TWI) derived from the DEM input in SAGA GIS (Conrad et al. 2015), following Kruse et al. (2022a). Water-covered grid cells were identified and masked in Google Earth Engine, using Sentinel 2’s band 8 near-infrared (Copernicus Sentinel data, ESA). Ignoring the grid cells containing water, elevation of the simulation area ranges between 106.1 and 122.7 m a.s.l. (mean = 113.8 m a.s.l.), slope values range between 0.1 and 4.5° (mean = 1.7°), and TWI values range between 7.1 and 15.5 (mean = 9.4).

In total, 25 different simulation scenarios were computed (for a structured overview, see Supplement 6). These include the simulations with and without the new wildfire module and those evaluating model sensitivity to input parameters, as described before. For the evaluation of fire return interval (FRI) and fire intensity (FI) impacts on forest structure, we simulated combinations of low (0.1), medium (0.5), and high fire intensity (1.0) at various fire return intervals (10, 50, 100, 200, and 300 years), resulting in 15 scenario-based simulation runs with fixed FRI and FI. For these scenarios, wildfires were set to affect the whole simulation area (i.e., in a scenario of low-intensity wildfires at an FRI of 50 years, the whole simulation area will be affected by a low-intensity fire every 50 years). Finally, two reference simulations using alternative climate forcing from MPI-ESM-CR (25,000 years BP to 2021 CE) and TraCE-21ka (22,000 years BP to 2021 CE) were computed.

### Statistical analyses of simulation output

LAVESI-FIRE was set to create a list of temporal output data (including, e.g., total stem count, mean tree height for trees > 200 cm, FPR_ann_, and number of burned cells) at annual resolution and write spatial output (including, e.g., the mean litter layer height, mean active layer depth, and tree abundance per species) for each individual 90 × 90 m grid cell of the simulation area every 100 years to restrict the size of total data output and computation time. Only grid cells that experienced a fire intensity larger than zero were included in the number of burned cells, so depending on the environmental conditions (i.e., high TWI), a grid cell within a burned area may be assigned a fire intensity of zero, thus excluding it from any fire impacts or from the number of burned cells.

To assess the number of mature trees, the stem count variable includes all trees ≥ 130 cm in height.

We applied a superposed epoch analysis in R (v.4.0.2; R Core Team 2020) to evaluate common responses of the forest to the various FRI and FI as applied in the 15 scenario-based simulation runs. Simulation data input was sorted using the “gtools” package (function “mixedsort()”; Bolker et al. 2022). Around each fire occurrence in a given simulation run, from the stem count timeseries 10 years pre- and 30 years post-fire were cut out. These snippets were superimposed to obtain a median response of each tree species for each scenario. Quantiles were determined using the “matrixStats” package (function “rowQuantiles()”; Bengtsson 2021). Line colors were derived from the “colorspace” package (function “qualitative_hcl()”; Zeileis et al. 2009, Zeileis et al. 2020). Spatial plots of the simulation area were done using the “lattice” package (function “levelplot”; Sarkar 2008).

## Results

### Sensitivity analysis

**Fig. 3:**
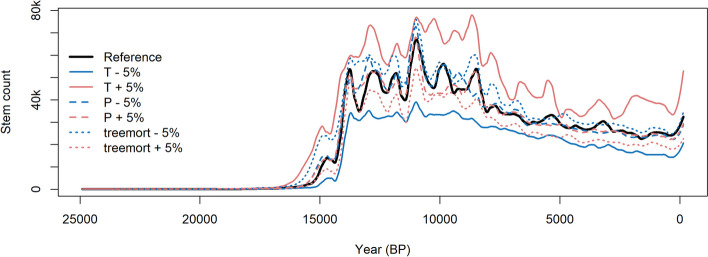
Sensitivity analysis, showing simulated total stem count of individual simulations (smoothed using a LOESS with a window width of 0.05)

Simulations for the sensitivity analysis, including an unchanged reference run, modified MPI-ESM1.2 climate input, and modified tree mortality, all followed a similar trend in the simulated forest development (Fig. 3). Runs with increased or decreased precipitation closely followed the unchanged reference run, indicating a minor influence on stem count when compared to the other changed variables. Runs with lower or higher tree mortality led to the expected outcome of a generally increased or decreased stem count, respectively. Runs with changed temperature deviated furthest from the reference run, indicating that temperature has the strongest impact on simulated stem count. Simulations with the alternative climate input from MPI-ESM-CR and TraCE-21ka depicted similar long-term trends to the main forcing data (Supplement 7).

### Fixed FRI and FI scenarios

At low fire intensity (0.1), there was no visible response of the total stem count after simulation area-wide fire occurrence of any for the tested FRI (Supplement 8). At a medium fire intensity (0.5) and at FRI = 50 years or higher, the number of Dahurian larches increased up to or above pre-fire numbers 10 to 20 years post-fire. Other tree species were non-existent or occurred only in very low numbers. At high fire intensity (1.0), effectively resetting the whole forest, tree abundance was greatly reduced. Dahurian larches ≥ 130 cm height started recovering from 5 years post-fire and, in case of frequent high-intensity fires at an FRI = 50 years, all tree species reached pre-fire numbers within 30 years. However, at FRI = 100 years or higher, the post-fire recovery of other species benefited more than that of the Dahurian larch*.* Other tree species such as Siberian larch, Siberian spruce, Scots pine, and Siberian pine could establish and were able to surpass their pre-fire numbers (Supplement 8). In general, fires of high intensity, resetting the population, tended to benefit trees besides Dahurian larch, whereas low-intensity fires were an advantage only for the relative share of the Dahurian larch.

**Fig. 4:**
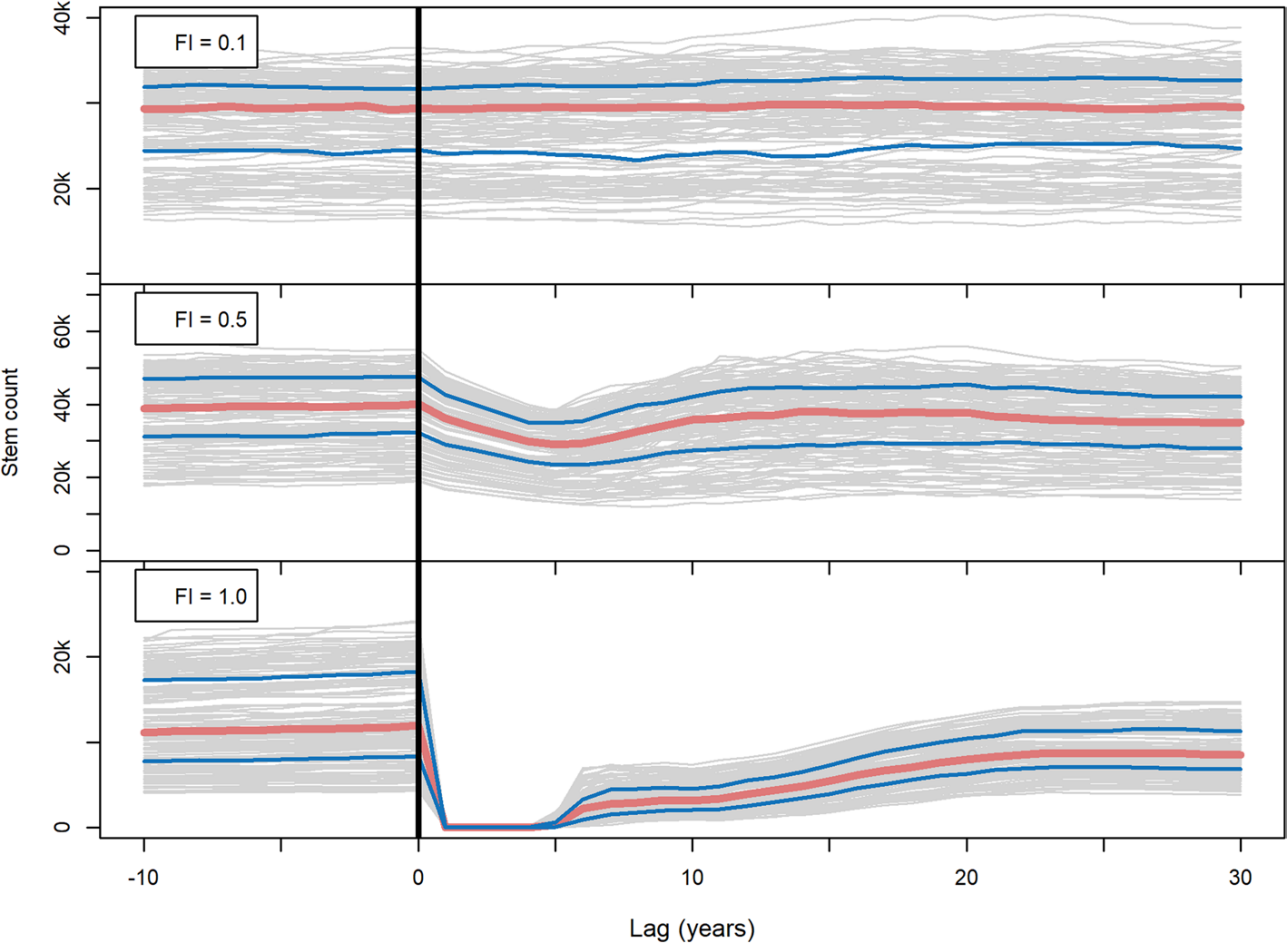
Superposed epoch analysis for compiled fire intensity (FI) scenarios. Black vertical line = year with fire occurrence; red line = median; blue lines = lower and upper quantiles. Gray lines represent the superimposed individual stem count timeseries that were cut out around each fire occurrence

Summarizing all trees within the simulated forest at an intermediate FRI = 100 years showed a similar outcome regarding different fire intensities (Fig. 4). Here, low-intensity wildfires (FI = 0.1) resulted in only a minor increase in tree mortality and thus did not leave an imprint on the stem count of the simulated forest (which does not include the more likely affected trees < 130 cm), regardless of the FRI. However, medium-intensity fires (FI = 0.5) led to an increase in trees 10 to 20 years later. High-intensity fires (FI = 1.0), on the other hand, resetting the population, resulted in greatly reduced tree abundance and were followed by a slow post-fire tree stand recovery phase, reaching pre-fire stem counts within 30 years or later (albeit with a different composition as Dahurian larch will be reduced and other species included in this stem count sum).

### Simulated fire activity and forest structure since the Last Glacial Maximum

**Fig. 5:**
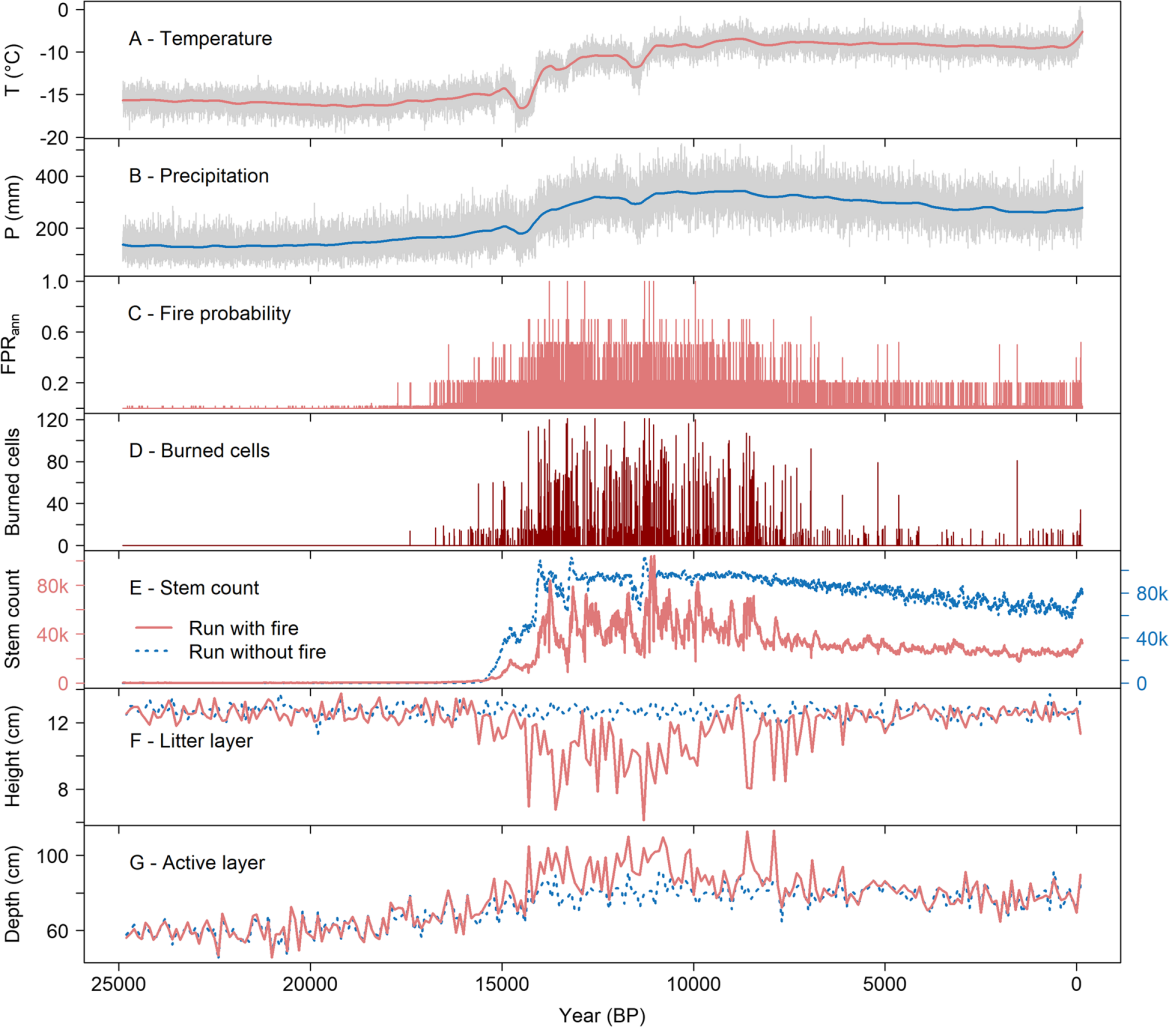
Timeseries of main simulation and reference run without fires. **A**, **B** Mean annual temperature and annual sum of precipitation, from MPI-ESM1.2. **C** Derived annual fire probability rating (FPR_ann_). **D** Annually burned grid cells within the simulation area. **E**–**G** Stem count, mean litter layer height, and mean active layer depth for the main simulation run with fire and the reference without fire, respectively. Note separate *y*-axes in plot (**E**)

In the simulation with climate-driven fire activity, modeled annual fire probability (FPR_ann_) was low during the Last Glacial Maximum (LGM; c. 20,000 years BP; Clark et al. 2009), increasing only after c. 17,000 years BP (Fig. 5). In the Late Glacial and Early to Mid-Holocene (c. 15,000 to 8000 years BP), extreme fire probability occurred more frequently than at any other time. From the Mid- to Late Holocene, FPR_ann_ remained at an intermediate level, before showing slightly increased values again towards the present. The number of burned cells within the simulation area follows this trend of FPR_ann_, with large fires taking place especially during the Early Holocene. The simulation-long mean FRI is 27 ± 50 years (mean ± 1σ), although no fire occurs before c. 17,000 years BP due to low FPR_ann_ after the LGM. When constrained to the Holocene, the mean FRI is 31 ± 52 years. The first half of the Holocene (11,700 to 6000 years BP) had a considerably shorter mean FRI (19 ± 91 years) when compared to the recent half (6000 years BP to present; 80 ± 91 years), although the mean FRI became shorter again in the most recent 150 years (11.5 ± 10 years).

In the simulation with fire, the total stem count for all tree species remained low during most of the Late Glacial, increasing sharply around the time of the Bølling-Allerød interstadial after c. 14,200 years BP. Around 11,600 years BP, a cooler and drier Younger Dryas period resulted in a c. 400 years long decrease in stem count. After returning to a high level, reaching its maximum around 11,000 years BP, stem count then gradually declined to low numbers in the Late Holocene.

The forest is clearly dominated by Dahurian larch; other species only occurred in low numbers. However, at the onset of favorable growing conditions in the Late Glacial (c. 16,000 years BP), Siberian larch managed to establish in elevated numbers during a c. 2000-year period, but were gradually outcompeted by the Dahurian larch population, which increased sharply after c. 14,000 years BP. Siberian spruce, Jack pine, and Siberian pine mostly managed to grow as seedlings only, with Cajander larch being the least abundant. Establishment is higher during periods of high fire activity for all species.

The mean litter layer height across the simulation area was c. 13 cm during the Late Glacial but started decreasing with more frequent fire activity and could completely disappear after simulation area-wide high-intensity wildfires. Due to a reduced insulation capacity of the burned litter layer, the mean active layer depth simultaneously increased from c. 60 cm during the Late Glacial to c. 100 cm during the Early Holocene. With less frequent fires in the Late Holocene, the mean litter layer could recover to c. 12 cm, with the active layer depth remaining at c. 80 cm. Both values correspond to field observations at Lake Satagay in 2018 CE (Kruse et al. 2019b).

**Fig. 6:**
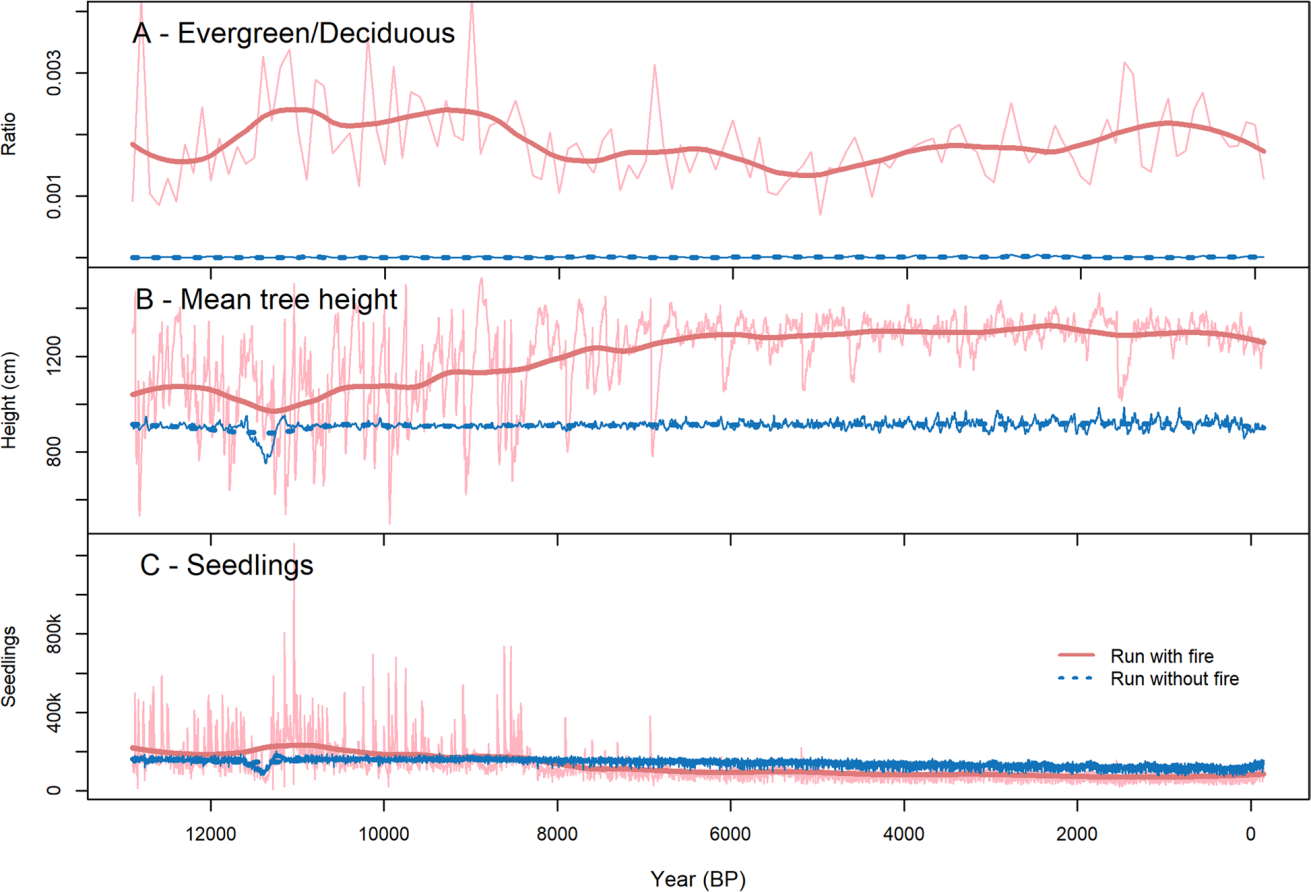
Forest structure as simulated with and without fire occurrence throughout the Holocene. **A** Ratio of evergreen to deciduous trees. **B** Mean tree height for mature trees > 200 cm. **C** Number of seedlings (trees between 0 and 40 cm)

Compared to a simulation without the fire module, the inclusion of wildfire disturbance strongly impacted simulated forest development. The added fire disturbance resulted in the forest fully establishing c. 1400 years later and with increased variability in tree abundance during the Late Glacial and Early Holocene (c. 14,200 to 8000 years BP). Fire occurrence prevented the forest from full establishment before c. 14,200 years BP, whereas without wildfires, rapid establishment preceded the Bølling-Allerød interstadial, occurring already at c. 15,600 years BP. Total stem count was reduced when wildfires could occur, whereas the variability within the number of trees was increased (Fig. 5). Only when wildfires were included did the ratio of evergreen to deciduous trees increase, especially in the Early and Late Holocene (Fig. 6A). The mean tree height was mostly constant at c. 900 cm in the run without fire, whereas fire inclusion resulted in increased decadal to centennial variability, more pronounced millennial-scale trends and generally increased tree height to about 1300 cm during the Late Holocene (Fig. 6B). Even though the stem count was lower in the simulation with fire, small seedlings (0 to 40 cm height) were more abundant in the Late Glacial and Early Holocene until c. 8000 years BP when compared to the simulation without fires (Fig. 6C).

## Discussion

### Wildfire impacts since the LGM

LAVESI-FIRE allows us to evaluate over a long timescale the annual impacts of introducing climate-driven fire disturbance to simulated tree population dynamics at a study site in the boreal forest of Central Yakutia, Siberia.

We find that frequent medium- to high-intensity fires, as seen in the Early Holocene, allow plenty of seedlings to establish, many of which would be consumed by a subsequent fire occurrence. Fires here act as a catalyst of tree establishment, which has been reported from previous studies and field observations (Tsvetkov 2004; Zyryanova et al. 2007; Kharuk et al. 2021; Miesner et al. 2022; Zhu et al. 2023). The main reason for this increase in establishment in the model is the reduction/removal of the litter layer, exposing soil and thus enabling seeds to germinate more frequently. The same effect of fires on germination rate has been experimentally demonstrated in Cajander larch forest of northeastern Yakutia (Alexander et al. 2018). Additionally, any fire damage on top of other mortality factors may lead to a faster removal of some older trees, creating a forest gap for new establishment. In reality, this effect is further supported by increased nutrient abundance after wildfires (e.g., phosphorus, potassium, and nitrogen; Kharuk et al. 2021). In addition, both in the field and in LAVESI-FIRE, tree mortality after wildfires reduces competition (Zyryanova et al. 2007). Since smaller trees and seedlings are more likely to sustain severe fire damage, taller trees (here mostly fire-adapted Dahurian larch) have an advantage, resulting in a generally increased mean tree height. In the field, decreased crown cover and competition for root space are additional reasons for an increase in post-fire growth (Zyryanova et al. 2007). In LAVESI-FIRE, the post-fire growth in height of individual trees is increased with a fire regime of infrequent, low- to medium-intensity fires (Mid to Late Holocene) and decreased with frequent high-intensity fires (Late Glacial and Early Holocene). Therefore, future fire regime intensification may result in generally decreased tree height and age in the Central Yakutian larch forests. Considering a correlation of tree height and the heat-insulating bark thickness, decreased stand ages may impact general wildfire resistance. Fire-related reductions of tree population ages have been confirmed in previous studies (Zyryanova et al. 2007; Kharuk et al. 2021; Zhu et al. 2023).

Our results indicate that post-fire regeneration of trees varies depending on the pre-fire forest structure. It may appear counter-intuitive that medium-intensity wildfires are often followed by increased tree establishment, whereas the initial establishment of a forest in the Late Glacial is slowed down by 1400 years when fires are simulated (Figs. 4E and 5). Fires in a mature forest can benefit growth of high trees and increase post-fire establishment, stabilizing the forested landscape, whereas frequent fires in open woodlands with few tall trees may instead prevent a forest from fully establishing in the first place. This relates to studies on fire-caused stable states and their potential tipping points (Lenton 2012; Scheffer et al. 2012), but may also point towards the existence of different post-fire regeneration pathways that depend on other factors. In our simulation, the discerning factor between a fire regime preventing full forest establishment and forests stabilized by the same fire regime may be the underlying climate-related growth conditions for the trees, meaning that in cooler and/or drier climate, fire may be more likely to act to prevent forest establishment and vice-versa. These contrasting post-fire regeneration pathways could potentially occur not only at different points in time, but also at different locations at the same time. For example, fire impacts of the same fire regime may be variable on a gradient from southern to northern Siberia.

The mean active layer depth is controlled by both long-term climate trends and local disturbances to the insulating organic layer, regulating heat fluxes. In LAVESI-FIRE, frequent high-intensity fires during the Late Glacial and Early Holocene result in a partial decoupling of the active layer depth from climatic trends. Instead, thawing depth during that time is mainly controlled by a ten-millennia-long general reduction of the insulating litter layer, due to the FRI being lower than the time needed for complete litter layer regeneration. The less severe fire regime of the Mid- to Late Holocene, in contrast, does not have this effect, as seen by a similar active layer depth in both simulations with and without fires (Fig. 5G). Although LAVESI-FIRE does not feature a detailed representation of permafrost processes, previous studies have emphasized the effect of changing fire regimes on the active layer depth (Knorre et al. 2019; Holloway et al. 2020; Petrov et al. 2022).

As observed in the field, Dahurian larch also clearly dominates the simulated forest at Lake Satagay (Kruse et al. 2019b; Miesner et al. 2022). Larches are adapted to frequent low-intensity fires with thick insulating bark, preventing cambium necrosis from flames heating the stem (Wirth 2005). The simulations suggest that a fire regime consisting of medium-intensity fires at an FRI ≥ 50 years not only increases the general stem count of the population, but benefits Dahurian larch dominance through increased post-fire establishment (Fig. 4B, Supplement 8). The dependence of Dahurian larch on frequent, low- to medium-intensity fires has been shown in previous studies (Tsvetkov 2004; Zyryanova et al. 2007) and is well reproduced within LAVESI-FIRE. In contrast, a fire regime of stand-replacing high-intensity fires results in vastly reduced stem counts and slow regeneration to pre-fire tree numbers (Fig. 4C, Supplement 8). Other tree species can establish under these cleared conditions. However, they remain at very low numbers. Currently near Lake Satagay there exist small populations of Scots pine on slightly elevated, dry and sandy soil patches, as well as small areas where larch grows mixed with Siberian spruce and Scots pine (Glückler et al. 2022). Due to a relatively coarse DEM at 90-m resolution and LAVESI-FIRE currently not representing such specific soil conditions, the establishment of pine or mixed forest patches in the model instead depends on a species’ climatic preferences, competitiveness, and tolerated minimum active layer depth. It seems that the last two factors prevent species other than Dahurian larch from establishing in higher numbers. For example, whereas Dahurian larch can grow at an active layer depth of ≥ 0.2 m, evergreen conifers require deeper thawing (1.0 m for Scots pine, 2.0 m for Siberian spruce and Siberian pine; Kruse et al. 2022a). This results in Dahurian larch generally establishing before other evergreen species, building a competitive advantage. In this case, other species can establish only when that competitive advantage is compromised. This is evident throughout the simulated stem count of the Holocene, where the inclusion of fire disturbance results in an increased ratio of evergreen to deciduous trees (Fig. 6A).

The trend of simulated FPR_ann_ and subsequent annually burned area throughout the Holocene is in good agreement with the sedimentary charcoal-based reconstruction of local wildfire activity at Lake Satagay for the past c. 10,800 years. Based on this reconstruction, it was hypothesized that open woodlands act as a positive feedback on wildfire activity, through increased fine fuel loads of grassy vegetation, faster drying of fuel from direct exposure to the sun, and higher wind speeds (Glückler et al. 2022). In both simulation and reconstruction, fire activity is highest in the Early Holocene before decreasing until c. 5000 years BP and remaining at comparatively low levels until the present day. However, in contradiction to the simulations, the pollen-based quantitative reconstruction of vegetation cover from the sediment core indicates an open forest landscape in the Early Holocene, growing gradually denser with decreasing fire activity (Glückler et al. 2022). Strongly decreased stem counts in the high fire intensity scenarios (Fig. 5) partially support the proposed relationship of open woodlands and intensified fire regimes.

Our results reinforce the findings by Stuenzi et al. (2022), where the coupled LAVESI-CryoGrid simulated different impacts on larch forest depending on the fire scenario (surface or canopy fires). Whereas surface fires increased the density of larch trees, the forest was not able to recover within 29 years after canopy fires. A similar conclusion was drawn by Shuman et al. (2017), simulating scenarios with fire disturbance across Russia in UVAFME. Since this relationship between Dahurian larch and wildfires is reproduced in various modeling studies and reinforced by empirical evidence, it appears likely that a continued intensification of fire regimes may reduce the species’ prevalence in eastern Siberia. In combination with rapidly warming temperatures and degrading permafrost that favors evergreen conifers (Herzschuh 2020), intensifying fire regimes may be an essential factor in determining the future forest structure and potential shifts from forest to steppe environments (Tchebakova et al. 2009; Scheffer et al. 2012). Therefore, an immediate goal within the fire-vegetation modeling community may be to narrow down a potential measurable threshold between stabilizing and destabilizing fire impacts in larch-dominated forests.

### Capability of LAVESI-FIRE

We find that the inclusion of wildfire disturbance has clear and variable impacts on long-term forest development and structure, mainly related to the total number of trees per species present and their height distribution. In this way, LAVESI-FIRE is able to demonstrate the impacts of introducing climate-driven wildfire disturbance on its simulated forest environment. As an individual-based model, it is setting a unique focus to evaluate fire impacts on tree populations undergoing full life cycles within a spatially explicit environment. This enables us to observe small-scale changes in forest structure and composition throughout the past 20,000 years and provides a perspective not present in larger-scale, more abstract modeling efforts.

The sensitivity analysis (Fig. 3) indicates the robustness of the simulated and discussed trends to changes in the climate input and fire-related mortality parameters. Furthermore, changes in temperature having the largest impact on simulated stem count when compared to the other variables are a result of the strong localization of LAVESI-FIRE. As a modification of the vegetation period, this outcome would be expected of a largely temperature-limited environment in Central Yakutia.

Simulation results should be viewed considering that FPR_ann_ is based on a linear model from climate data to satellite-derived burned area between 2001 and 2021 CE. The relationship between temperature, precipitation, and fire probability is thus constant through time and based on values of the present year only. However, weather conditions of the previous year may also influence fire probability (Wang et al. 2021). The importance of non-static disturbance modules within models simulating long-term vegetation development has recently been highlighted by Dallmeyer et al. (2023), who report a mismatch between reconstructed and simulated tree cover in Europe throughout the Holocene. Furthermore, it is debatable to what degree humans interfered in the burned area observed during those past 20 years, and what impact such interference may have on the long-term climate-fire relationship. For example, landscape fragmentation may today artificially limit fire extent, and fires close to settlements or infrastructure are actively suppressed. Solovyeva et al. (2020) further mention a centuries-old tradition of agricultural burning and the collection of deadwood and litter to reduce fuel loads in Central Yakutia. Such impacts on fire activity are difficult to quantify in retrospect. However, since the purpose of this study is not to achieve a factual quantification of fire regime impacts since the LGM, but rather to analyze systematic relationships between changes in fire regimes and forest structure, we argue that the human component, in both parameter tuning as well as simulation outcome, does not meaningfully change the reported findings.

In its current state, LAVESI-FIRE simulates only one fire per annual simulation timestep, depending on FPR_ann_. For local simulation areas a few kilometers in diameter this is sufficient, but if the simulation area were to be increased to a regional scale, implementing multiple ignitions per timestep may be appropriate. Local-scale simulations, as presented in this study, may also benefit from a higher-resolution DEM input, possibly even using a fine-scale LiDAR-derived DEM to capture microtopography and related effects on fuel moisture.

For approximating long-term fire impacts over multiple millennia, we omitted the inclusion of active fire spread. While we suggest that the relationship between annual fire probability and burned area is a valid simplification to evaluate the research objectives of this study, LAVESI-FIRE does not currently simulate overwintering fires, although they may add significantly to the annual burned area with sustained fire regime intensification (Xu et al. 2022). This is in part a consequence of omitting fire spread and a related differentiation between running and sustained surface fires (Kharuk et al. 2021). Running surface fires, which can be considered the standard in LAVESI-FIRE under low FPR_ann_, will generally result in lower mortality of mature trees than sustained surface fires. The latter tend to burn deeper into the organic layer and soil and thus result in increased tree mortality by heating the permafrost-limited rooting space (Sofronov and Volokitina 2010; Alexander et al. 2018; Bär et al. 2019). The present version of LAVESI-FIRE provides a foundation to implement and refine these contrasting surface fire regimes, and thus enable new simulation scenarios and research objectives in the future.

While the actual fire impacts are mediated by tree attributes (total height, relative crown height, bark thickness, etc.) and ground conditions such as estimated moisture availability, other processes such as fuel quantification have been omitted here. We suggest that for long-term simulations at annual resolution, our applied relationships between climate-driven fire probability and total burned area are sufficient. However, in order to test for complete fire-vegetation feedback, an additional implementation of fuel impacts on fire intensity and size may be needed. Recent evidence from the North American boreal forest points towards a significant contribution of fuel availability to fire severity (Walker et al. 2020), even though it remains to be evaluated whether this applies to eastern Siberia. Dynamic fuel-fire interactions may enable the model to examine more closely questions related to stable states and tipping points in forest structure instead of strictly unidirectional impacts of changing fire regimes on the forest.

Apart from the aforementioned LAVESI-Cryogrid (Stuenzi et al. 2022), the only comparable individual-based fire-vegetation model other than LAVESI-FIRE recently applied in Siberia is the forest gap model UVAFME (Shuman et al. 2017). Although some basic aspects of fire implementation are similar between the two models, they work very differently and thus fulfill different purposes, each with their own advantages. For example, LAVESI-FIRE includes a spatially explicit environment derived from a custom DEM input at the study site, whereas UVAFME, as a forest gap model, simulates small 500 m^2^ plots that are described as being spatially homogeneous. While LAVESI-FIRE is therefore able to simulate the actual environment for wildfires to act upon, the individual plots of UVAFME are less computationally demanding and are set up to be applied on a broader scale (i.e., to cover all of Russia; Shuman et al. 2017). Furthermore, fire frequency in UVAFME is determined from remote sensing data and translated into a temporally fixed fire probability, only mediated by aridity. In LAVESI-FIRE the fire probability is directly linked to monthly estimated, variable fire weather conditions, enabling fire regime changes. Scenario-based simulations limited to the inclusion or exclusion of fires may be unable to capture non-linear responses of larch trees to changing fire regimes (Kharuk et al. 2021). Another difference is the inclusion of the litter layer in LAVESI-FIRE, which we find here is an important factor in how wildfires affect tree establishment. Despite these differences, both models demonstrate how individual-based modeling provides a valuable, fine-scale, and highly localized perspective on fire-vegetation interactions.

Going forward, updating the model with pyrogenic carbon production and spread, which could be readily linked with the already implemented pollen dispersal module, would enable it to simulate sedimentary charcoal records to be compared to paleoecological reconstructions. Fire regime attributes and drivers necessary to produce a given record of sedimentary fire proxies could be better understood by tuning the model outputs to the reconstructed data. Finally, LAVESI-FIRE may be well suited to analyze and estimate quantitative anthropogenic impacts on wildfire regimes, for example, effects of historical agricultural burning or the modern degree of landscape fragmentation by implementing artificial fire breaks. Since human impacts are among the most challenging to quantify and disentangle from climate and vegetation in long-term paleoecological fire studies (Marlon et al. 2013), this could provide valuable insights to benefit reconstructions of the past and thus improve simulations of the future.

## Conclusions

In this study, we aimed to evaluate the long-term impacts of climate-driven fire disturbance on forest structure in Central Yakutia, eastern Siberia. For this, the individual-based vegetation model LAVESI was extended with a new wildfire module and run to simulate forest development with fire disturbances at a local study site since the LGM. Simulation results in LAVESI-FIRE show that the inclusion of wildfires has variable impacts on forest structure throughout the last c. 20,000 years and differs from a reference simulation without fire in many ways. While total tree abundance decreases, mean tree height increases, likely due to reduced competition. The forest fully establishes c. 1400 years later, and in the Late Glacial and Early Holocene, high numbers of seedlings can temporarily establish between fires due to a decreased litter layer. Under a similar fire regime, post-fire forest regeneration may follow different pathways, depending on environmental and climatic conditions. Whereas medium-intensity fires at a frequency of 50 or more years improve Dahurian larch growing conditions, stand-replacing high-intensity fires are followed by a slower and only partial regeneration of the Dahurian larch population, enabling other evergreen species to establish in low numbers. These results highlight both the value of long-term simulations, as well as the importance of including wildfire disturbance when simulating long-term forest development. As they are not merely destructive events, but result in a non-constant modification of landscapes, wildfire disturbance is required to fully understand past and future environmental changes.

### Supplementary Information

**Additional file 1: Supplement 1.** Estimation of monthly fire probability rating (FPR_mon_) based on monthly mean temperature (T_mon_) and precipitation (P_mon_). **Supplement 2.** (Left) Linear model for predicting fire probability from T and P compared to observed fires from MODIS. (Right): Modelled FPR_mon_ values for months without observed fires. To limit false-positive fire probability in the model, Q4 = 6.6 was used as minimum threshold for assigning fire probability to a given month. **Supplement 3.** (Left) of FPR_mon_ above the minimum threshold (6.6), showing in red the separations of mild, severe and extreme fire probability thresholds. (Right): Boxplot of the same FPR_mon_ values, indicating how thresholds for the monthly categorization of fire weather were chosen (for severe fire: Q3 = 7.0, for extreme fire: Q4 = 7.46). **Supplement 4.** Estimation of annual fire probability rating (FPR_ann_). **Supplement 5.** Estimation of topographic wetness index (TWI) mediating impact. **Supplement 6.** Overview of the different simulation scenarios. Climate forcing #1 is the main MPI-ESM1.2 forcing data, #2 and #3 are the alternative forcing datasets from MPI-ESM-CR and TraCE-21ka, respectively. **Supplement 7.** Alternative climate forcing model data and corresponding simulated stem count. **Supplement 8.** Superposed epoch analysis for selected FRI/FI scenarios, showing the stem count median per species for fire occurrences after 14,000 yrs BP.

## Data Availability

The datasets generated and analyzed in this study are available in the Zenodo repository (Glückler and Kruse 2023; 10.5281/zenodo.10183691). The LAVESI-FIRE model code, including the input data used in this study, can be accessed via GitHub (https://github.com/StefanKruse/LAVESI/tree/fire).
