# Supplementary material for: Simulating long-term wildfire impacts on boreal forest structure in Central Yakutia, Siberia, since the Last Glacial Maximum
Source: Fire Ecol. 2024 Jan 4;20(1):1. doi: 10.1186/s42408-023-00238-8 (PMC10766680; doi:10.1186/s42408-023-00238-8)
Supplement: Supplementary file 1 — Additional file 1: Supplement 1. Estimation of monthly fire probability rating (FPRmon) based on monthly mean temperature (Tmon) and precipitation (Pmon). Supplement 2. (Left) Linear model for predicting fire probability from T and P compared to observed fires from MODIS. (Right): Modelled FPRmon values for months without observed fires. To limit false-positive fire probability in the model, Q4 = 6.6 was used as minimum threshold for assigning fire probability to a given month. Supplement 3. (Left) of FPRmon above the minimum threshold (6.6), showing in red the separations of mild, severe and extreme fire probability thresholds. (Right): Boxplot of the same FPRmon values, indicating how thresholds for the monthly categorization of fire weather were chosen (for severe fire: Q3 = 7.0, for extreme fire: Q4 = 7.46). Supplement 4. Estimation of annual fire probability rating (FPRann). Supplement 5. Estimation of topographic wetness index (TWI) mediating impact. Supplement 6. Overview of the different simulation scenarios. Climate forcing #1 is the main MPI-ESM1.2 forcing data, #2 and #3 are the alternative forcing datasets from MPI-ESM-CR and TraCE-21ka, respectively. Supplement 7. Alternative climate forcing model data and corresponding simulated stem count. Supplement 8. Superposed epoch analysis for selected FRI/FI scenarios, showing the stem count median per species for fire occurrences after 14,000 yrs BP. [file 42408_2023_238_MOESM1_ESM.docx]

**Simulating long-term wildfire impacts on boreal forest structure in Central Yakutia,
Siberia, since the Last Glacial Maximum**

**Supplement**

Additional formulas and figures related to the implementation of fire occurrence in LAVESI-FIRE are shown here. For the full code or simulation data, please refer to “Availability of data and materials” in the main research paper.

**Supplement 1:** Estimation of monthly fire probability rating (FPR_mon_) based on monthly mean temperature (T_mon_) and precipitation (P_mon_).

$${FPR}_{mon}=(0.11816\times PC1+(-0.12372\times PC2))+1.46818$$

$$PC1=\left( T_{mon}+12.03 \right)\times0.7213351+\left( P_{mon}-39.14 \right)\times0.6925862$$

$$PC2=\left( T_{mon}+12.03 \right)\times(-0.6925862)+\left( P_{mon}-39.14 \right)\times0.7213351$$

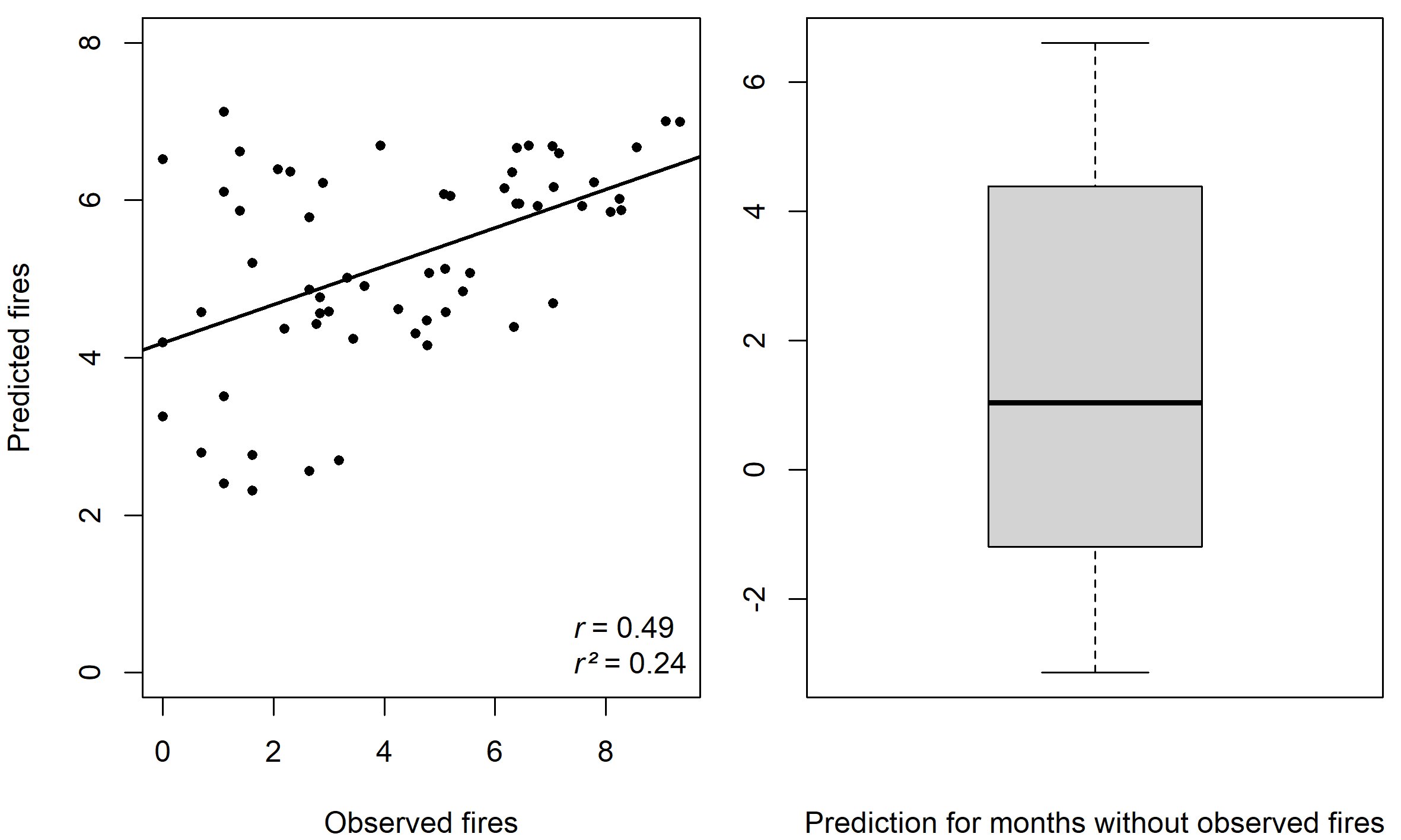


**Supplement 2:** (Left) Linear model for predicting fire probability from T and P compared to observed fires from MODIS. (Right): Modelled FPR_mon_ values for months without observed fires. To limit false-positive fire probability in the model, Q4 = 6.6 was used as minimum threshold for assigning fire probability to a given month.


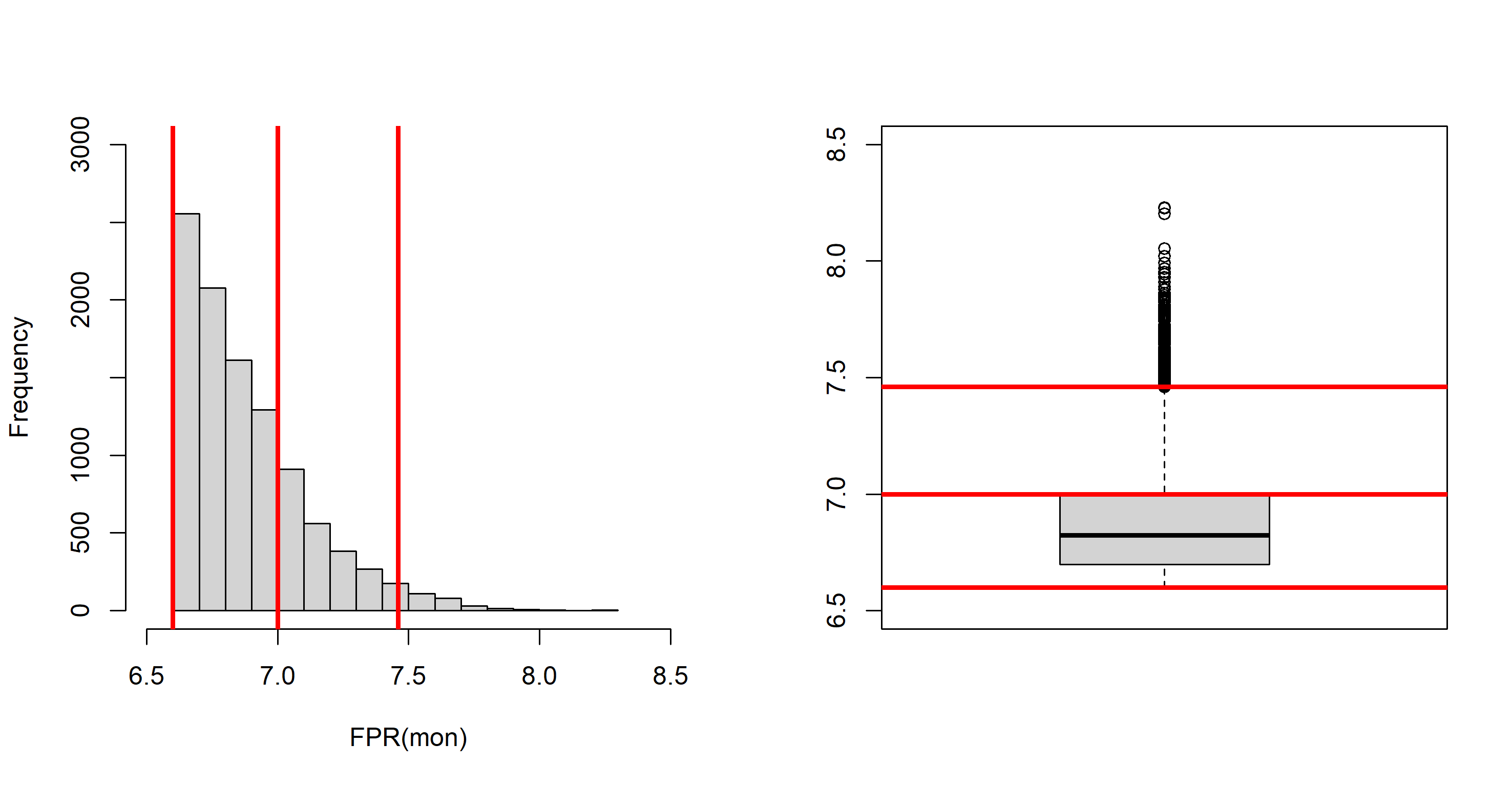


**Supplement 3:** (Left) of FPR_mon_ above the minimum threshold (6.6), showing in red the separations of mild, severe and extreme fire probability thresholds. (Right): Boxplot of the same FPR_mon_ values, indicating how thresholds for the monthly categorization of fire weather were chosen (for severe fire: Q3 = 7.0, for extreme fire: Q4 = 7.46).

**Supplement 4:** Estimation of annual fire probability rating (FPR_ann_).

$${FPR}_{ann}=n_{mild}\times0.02+\left. n_{severe}\times0.2 \right.+n_{extreme}\times0.5$$

**Supplement 5:** Estimation of topographic wetness index (TWI) mediating impact.

$$Fire intensity = {FPR}_{ann}^{\left( TWI\div100 \right)\div12.5}$$

**Supplement 6:** Overview of the different simulation scenarios. Climate forcing #1 is the main MPI-ESM1.2 forcing data, #2 and #3 are the alternative forcing datasets from MPI-ESM-CR and TraCE-21ka, respectively. For references, please refer to the main research paper.

| **Simulation ID** | **Climate forcing** | **FRI changes** | **FI changes** | **Parameter changes** |
| --- | --- | --- | --- | --- |
| sim_1 | 1 | Climate-driven | Climate-driven | - |
| sim_2 | 1 | No fire occurrence | No fire occurrence | - |
| sim_3 | 1 | - | - | T - 5% |
| sim_4 | 1 | - | - | T + 5% |
| sim_5 | 1 | - | - | P - 5% |
| sim_6 | 1 | - | - | P + 5% |
| sim_7 | 1 | - | - | fire mortality - 5% |
| sim_8 | 1 | - | - | fire mortality + 5% |
| sim_9 | 1 | 10 | Low (0.1) | - |
| sim_10 | 1 | 50 | Low (0.1) | - |
| sim_11 | 1 | 100 | Low (0.1) | - |
| sim_12 | 1 | 200 | Low (0.1) | - |
| sim_13 | 1 | 300 | Low (0.1) | - |
| sim_14 | 1 | 10 | Medium (0.5) | - |
| sim_15 | 1 | 50 | Medium (0.5) | - |
| sim_16 | 1 | 100 | Medium (0.5) | - |
| sim_17 | 1 | 200 | Medium (0.5) | - |
| sim_18 | 1 | 300 | Medium (0.5) | - |
| sim_19 | 1 | 10 | High (1.0) | - |
| sim_20 | 1 | 50 | High (1.0) | - |
| sim_21 | 1 | 100 | High (1.0) | - |
| sim_22 | 1 | 200 | High (1.0) | - |
| sim_23 | 1 | 300 | High (1.0) | - |
| sim_24 | 2 | Climate-driven | Climate-driven | - |
| sim_25 | 3 | Climate-driven | Climate-driven | - |


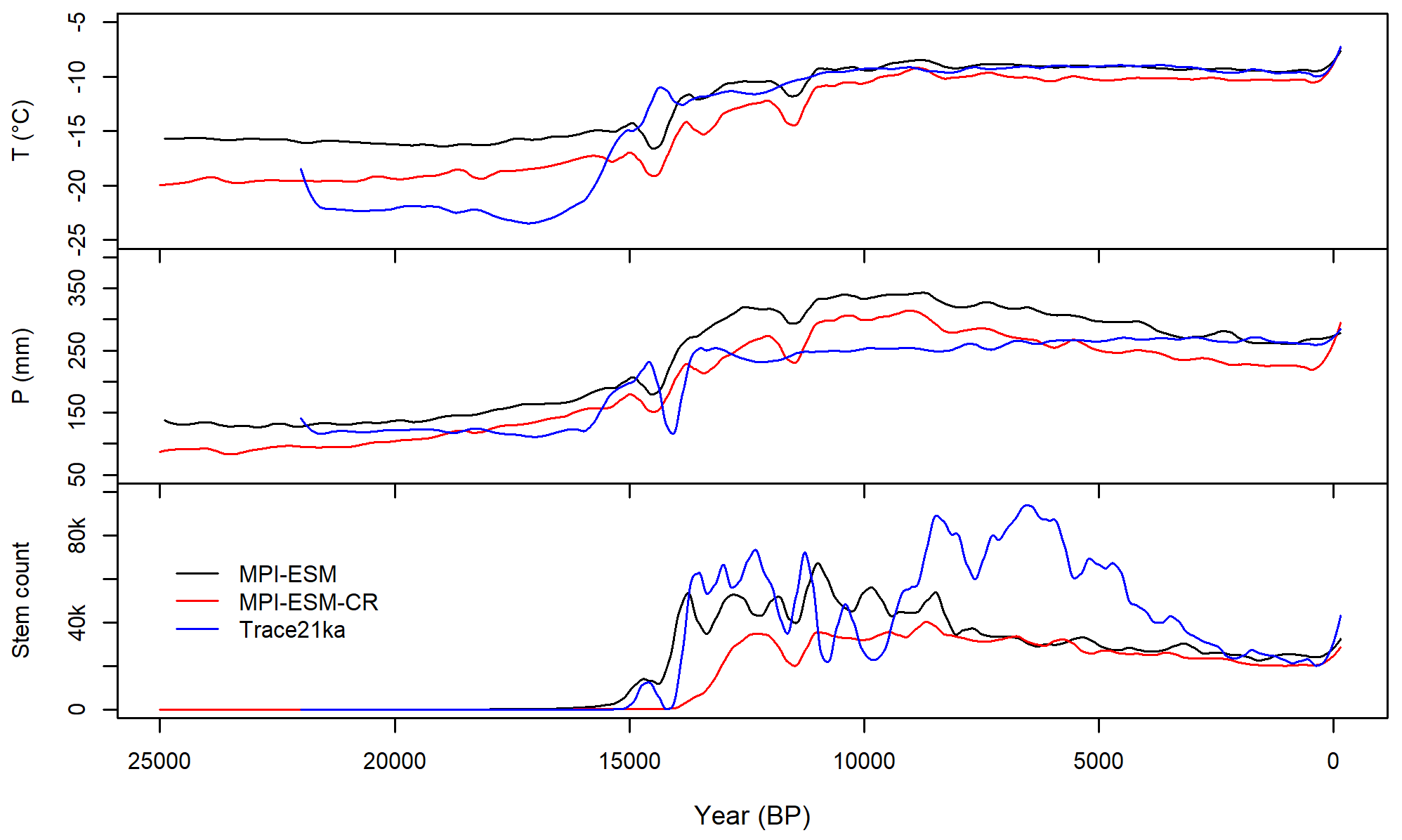


**Supplement 7:** Alternative climate forcing model data and corresponding simulated stem count.


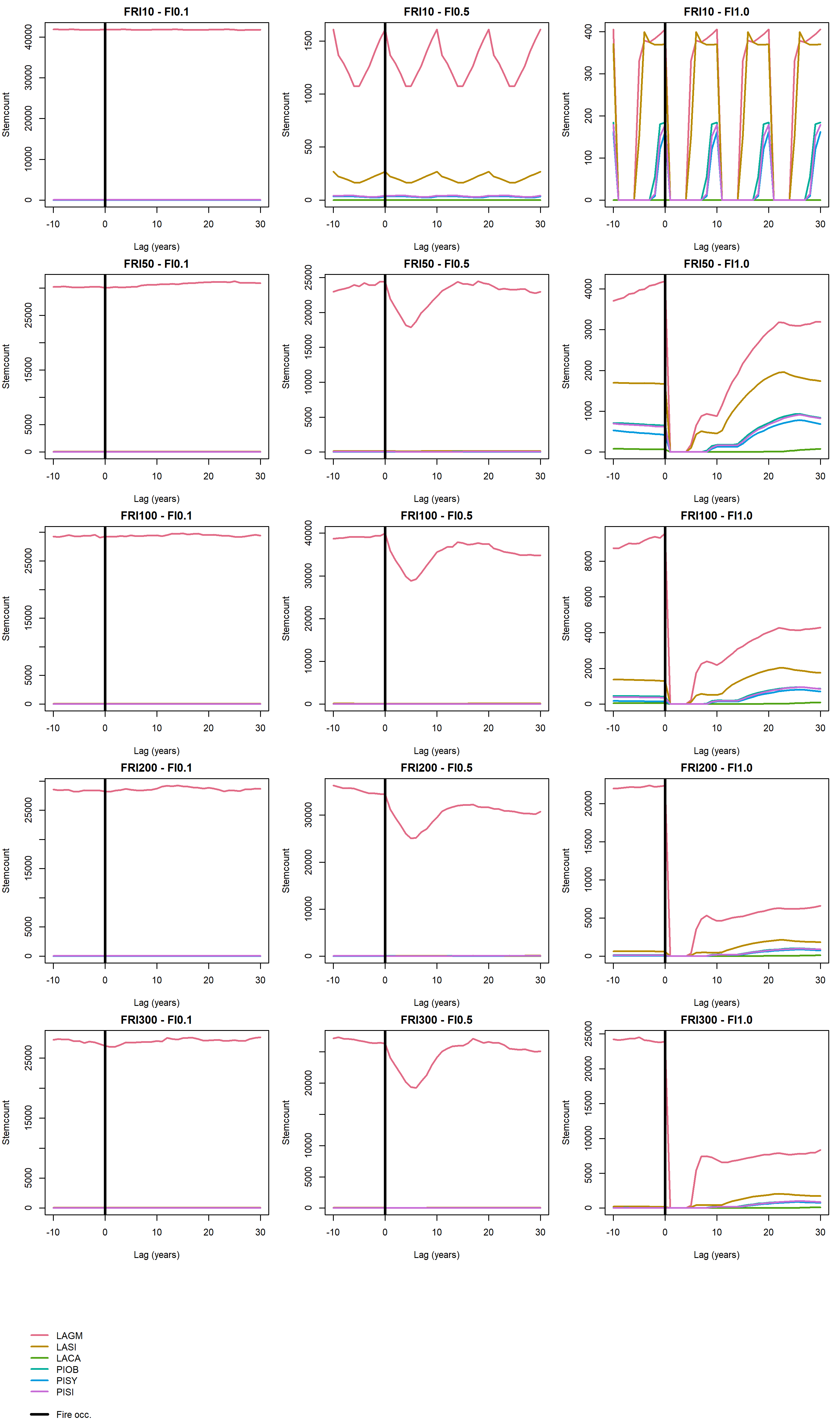


**Supplement 8:** Superposed epoch analysis for selected FRI/FI scenarios, showing the stem count median per species for fire occurrences after 14,000 yrs BP.
